# Life on Arginine for *Mycoplasma hominis*: Clues from Its Minimal Genome and Comparison with Other Human Urogenital Mycoplasmas

**DOI:** 10.1371/journal.pgen.1000677

**Published:** 2009-10-09

**Authors:** Sabine Pereyre, Pascal Sirand-Pugnet, Laure Beven, Alain Charron, Hélène Renaudin, Aurélien Barré, Philippe Avenaud, Daniel Jacob, Arnaud Couloux, Valérie Barbe, Antoine de Daruvar, Alain Blanchard, Cécile Bébéar

**Affiliations:** 1 Université de Bordeaux, Laboratoire de Bactériologie EA 3671, Bordeaux, France; 2 INRA, UMR 1090, Villenave d'Ornon, France; 3 Université de Bordeaux, UMR 1090, Villenave d'Ornon, France; 4 Université de Bordeaux, Centre de Bioinformatique de Bordeaux, Bordeaux, France; 5 CNRS UMR 5800, Laboratoire Bordelais de Recherche en Informatique, Talence, France; 6 Génoscope, Centre National de Séquençage, Evry, France; Universidad de Sevilla, Spain

## Abstract

*Mycoplasma hominis* is an opportunistic human mycoplasma. Two other pathogenic human species, *M. genitalium* and *Ureaplasma parvum*, reside within the same natural niche as *M. hominis*: the urogenital tract. These three species have overlapping, but distinct, pathogenic roles. They have minimal genomes and, thus, reduced metabolic capabilities characterized by distinct energy-generating pathways. Analysis of the *M. hominis* PG21 genome sequence revealed that it is the second smallest genome among self-replicating free living organisms (665,445 bp, 537 coding sequences (CDSs)). Five clusters of genes were predicted to have undergone horizontal gene transfer (HGT) between *M. hominis* and the phylogenetically distant *U. parvum* species. We reconstructed *M. hominis* metabolic pathways from the predicted genes, with particular emphasis on energy-generating pathways. The Embden–Meyerhoff–Parnas pathway was incomplete, with a single enzyme absent. We identified the three proteins constituting the arginine dihydrolase pathway. This pathway was found essential to promote growth *in vivo*. The predicted presence of dimethylarginine dimethylaminohydrolase suggested that arginine catabolism is more complex than initially described. This enzyme may have been acquired by HGT from non-mollicute bacteria. Comparison of the three minimal mollicute genomes showed that 247 CDSs were common to all three genomes, whereas 220 CDSs were specific to *M. hominis*, 172 CDSs were specific to *M. genitalium*, and 280 CDSs were specific to *U. parvum*. Within these species-specific genes, two major sets of genes could be identified: one including genes involved in various energy-generating pathways, depending on the energy source used (glucose, urea, or arginine) and another involved in cytadherence and virulence. Therefore, a minimal mycoplasma cell, not including cytadherence and virulence-related genes, could be envisaged containing a core genome (247 genes), plus a set of genes required for providing energy. For *M. hominis*, this set would include 247+9 genes, resulting in a theoretical minimal genome of 256 genes.

## Author Summary

*Mycoplasma hominis*, *M. genitalium*, and *Ureaplasma parvum* are human pathogenic bacteria that colonize the urogenital tract. They have minimal genomes, and thus have a minimal metabolic capacity. However, they have distinct energy-generating pathways and distinct pathogenic roles. We compared the genomes of these three human pathogen minimal species, providing further insight into the composition of hypothetical minimal gene sets needed for life. To this end, we sequenced the whole *M. hominis* genome and reconstructed its energy-generating pathways from gene predictions. Its unusual major energy-producing pathway through arginine hydrolysis was confirmed in both genome analyses and *in vivo* assays. Our findings suggest that *M. hominis* and *U. parvum* underwent genetic exchange, probably while sharing a common host. We proposed a set of genes likely to represent a minimal genome. For *M. hominis*, this minimal genome, not including cytadherence and virulence-related genes, can be defined comprising the 247 genes shared by the three minimal genital mollicutes, combined with a set of nine genes needed for energy production for cell metabolism. This study provides insight for the synthesis of artificial genomes.

## Introduction

*Mycoplasma hominis* is an opportunistic human mycoplasma species which resides, as a commensal, in the lower urogenital tract. However, it can also cause pelvic inflammatory disease and postpartum or postabortion fevers, and has been associated with bacterial vaginosis [1]. In newborns, it can cause pneumonia, meningitis or abscesses. It has also been implicated in extragenital infections, especially in immunocompromised patients.

Two other mollicute species, *M. genitalium* and *Ureaplasma urealyticum*, the latter having been recently separated into two species *U. parvum* and *U. urealyticum* [2], have established pathogenic roles in humans and reside within the same natural niche, the urogenital tract, as *M. hominis*. However, these species have overlapping but distinct pathogenic roles [1]. *M. genitalium* and *Ureaplasma* spp., but not *M. hominis*, are involved in male urethritis. *M. genitalium* is also the only mycoplasmal species involved in cervicitis, whereas *Ureaplasma* spp. are significantly associated with prematurity, low birth weight and chronic lung disease in infants [1]. These three urogenital species belong to two different phylogenetic groups within the class *Mollicutes*: *M. genitalium* and *Ureaplasma* spp. belong to the Pneumoniae group and *M. hominis* belongs to the Hominis group. They possess the smallest genomes among self-replicating free living organisms. The *M. genitalium* genome is the smallest of these, comprising 580 Kbp, with a capacity to encode only 482 genes [3]. The minimal nature of the *M. genitalium* genome triggered particular interest in this organism and several studies have addressed the concept of a minimal cell [4]–[6]. More recent studies have attempted to reconstruct its genome by chemical synthesis [7],[8], with a view to engineering a new living organism, referred to as *Mycoplasma laboratorium* [9]. The genome of *M. hominis* is slightly larger than that of *M. genitalium*, with pulsed-field gel electrophoresis revealing a chromosome size of 696 Kbp [10]. The sequenced genome of *U. parvum* (previously called *U. urealyticum* serovar 3) is the largest of the three species, encompassing 751 Kbp [11]. These three species can therefore be considered as minimal bacterial cell prototypes with reduced metabolic abilities. Interestingly, in addition to their distinct pathogenic roles, they also have different energy-generating pathways. *M. genitalium* is a glycolytic species, whereas *M. hominis* and *Ureaplasma* spp. are both nonglycolytic species, producing energy through arginine degradation or urea hydrolysis, respectively [12]. Thus, during the evolution of mollicutes, these three human pathogens have undergone substantial genome reduction resulting in minimal, but distinct, metabolic mechanisms.

Of all the mycoplasma species with a known pathogenic role in humans, *M. hominis* was the only one that had not been sequenced. We therefore sequenced the whole genome of the *M. hominis* PG21 type strain. We examined its energy-generating pathways and investigated the essential role of its arginine dihydrolase pathway *in vivo*. To provide further insight into the composition of hypothetical minimal gene sets and of the associated energy-generating pathways, we carried out whole genome comparisons of the three pathogenic mycoplasmas, *M. hominis*, *M. genitalium* and *U. parvum*, sharing the same urogenital niche and displaying near minimal genomes. This analysis resulted in the identification of a set of shared genes representing the core genome. Other genes include those that are involved in the energy-yielding pathways specific to each mollicute and those that play a role in the interaction with the host, such as cytadherence and virulence.

## Results

### General features of the *M. hominis* genome

The general features of the *M. hominis* PG21 genome and their comparison with those of *M. genitalium* G37 and *U. parvum* serovar 3 are shown in Table 1. The *M. hominis* PG21 genome is a single, circular chromosome of 665,445 bp with an overall G+C content of 27.1%. It contains 537 putative coding DNA sequences (CDSs), representing a 89.8% gene density, and 14 pseudogenes were found. Function could be predicted for 345 of the CDSs, whereas 86 were conserved hypothetical proteins (CHP) and 106 were hypothetical proteins (HP). A minimal but complete set of 33 tRNA genes was identified. The *M. hominis* genome contained two copies of rRNA genes, as previously described [10]. The 5S rRNA genes are not located within the 16S–23S rRNA operons, as is the case in *M. arthritidis* [13], the phylogenetically closest genome-sequenced species. No insertion sequence, transposon, or endogenous plasmid was found in the genome.

**Table 1:** Comparison of general features of the *M. hominis* genome with those of *M. genitalium* G37 (ATCC 33530) and *U. parvum* serovar 3 (ATCC 700970).

Features^a^	*M. hominis* PG21	*M. genitalium* G37	*U. parvum serovar* 3
Genome size (bp)	**665,445**	580,074	751,719
GC content of genome (%)	**27.1**	31.7	25.5
Gene density (%)	**89.8**	91.2	91.3
Total number of CDS	**537**	482	614
Hypothetical proteins	**106**	2	182
Conserved hypothetical proteins	**86**	164	107
CDSs with predicted fonction	**345**	316	325
Pseudogenes	**14** b	5^c^	0^c^
Average protein length (aa)	**369**	363	371
Proteins with predicted MW>300 kDa	**2**	0	2
Predicted lipoproteins	**43**	18^d^	37^d^
rRNA sets	**2** e	1	2
tRNA	**33**	36	30
Start codon usage (%)			
AUG	**95.1**	88.5	92.5
UUG	**3.0**	4.1	3.0
GUG	**1.7**	7.4	4.5
Other	**0.2**	0	0
Stop codon usage (%)			
UAA	**83**	72	85
UAG	**16**	27	14
Tryptophan codon usage (%)			
UGA	**87**	64	87
UGG	**13**	36	13

a Previously published data [3],[11], not including average protein length, the high molecular weight protein prediction, and start and stop codon usage, obtained from the MolliGen website (http://cbi.labri.fr/outils/molligen).

b Each part of a pseudogene is counted.

c Data from the Integrated Microbial Genomes website (http://imgweb.jgi-psf.org/cgi-bin/w/main.cgi?page=home).

d Lipoprotein number based on genome annotation.

e Two sets of 16S–23S rRNA genes and two single 5S rRNA genes elsewhere in the genome.

CDS, coding sequence; ND, not determined; MW, molecular weight.

A set of 43 lipoproteins was predicted from the *M. hominis* genome, including three ABC transporter substrate-binding proteins (MHO_3610, MHO_3620, MHO_1510), two predicted nucleases (MHO_0660, MHO_0730) and one predicted peptidase (MHO_4970). Interestingly, the lipobox sequence, located upstream from the cleavage site, was highly conserved in five of the predicted lipoproteins, MHO_1730, MHO_2100, MHO_2340, MHO_2440, MHO_2620, with the consensus motif PLVAAGC present in all of them. In contrast to these conserved regions, the other regions of the proteins were very different in length and sequence, suggesting their rapid and divergent evolution from a common ancestral gene.

We assigned the origin of replication based on sequence homology with the *M. arthritidis* genome [13] and several other mycoplasmas [14] for which this *oriC* was experimentally demonstrated to be functional. We did not detect any significant inversion in the GC skew for *M. hominis*. The synteny *rnpA *→ *rpmH *→ *dnaA *→ *dna*N was present, as previously observed in *M. arthritidis*, *M. capricolum* subsp. *capricolum* and *M. mycoides* subsp. *mycoides* SC [13]–[15]. Two identical putative DnaA boxes (TTATTAACA) were found in the intergenic region upstream of the *dnaA* gene, but none between *dnaA* and *dna*N. These boxes showed sequence identity at seven of the nine positions of the *Escherichia coli* consensus sequence TTATCCACA [16]. They were also identical to the unique DnaA box upstream from the *dnaA* gene in *M. mobile* [17] and *M. arthritidis* [13], both of which belong to the same phylogenetic group as *M. hominis*.

### Horizontal gene transfers from mollicutes sharing the same ecological niche

We identified genes that may have been exchanged through horizontal gene transfer (HGT), by searching for *M. hominis* CDSs that show a best Blast hit (BBH) in species other than those belonging to the Hominis phylogenetic group. From the 537 CDSs predicted from the *M. hominis* chromosome, 59 had a BBH in a mollicute belonging to the phylogenetic groups Pneumoniae, Spiroplasma or Phytoplasma and 12 had a non-mollicute BBH. We then examined each of the candidate CDSs for potential HGT, using phylogenetic reconstructions and synteny analysis. Five CDSs seemed to be likely candidates for HGT between *M. hominis* and non-mollicute bacteria (Table 2). Their closest homologs were found in commensal or pathogenic bacteria previously identified in humans, suggesting a possible scenario for gene exchange. One of the five CDSs (MHO_2540) was of particular interest because it encoded a putative N-dimethylarginine dimethylaminohydrolase (DDAH), an enzyme linked to the arginine pathway (see below). Among other mollicutes, only one clear orthologous gene was predicted in the phylogenetically closest related *M. arthritidis* (85% similarity), but the deduced protein was truncated in N-terminal by a third of its length. A putative DDAH was also proposed in *M. penetrans* (MYPE1510) but it appeared very different from MHO_2540 (45% similarity) and from other bacterial orthologs, suggesting a specific evolution and/or origin. Except in *M. arthritidis*, closest homologs of MHO_2540 were found in non-mollicute bacteria including the human pathogens *Eggerthella lenta* (76% similarity), *Atopobium vaginae* (72% similarity) and *Pseudomonas aeruginosa* (71% similarity). Although phylogenetic reconstructions remained unclear, this suggests that MHO_2540 and its truncated ortholog in *M. arthritidis* might have a non-mollicute origin.

**Table 2:** Horizontal transfer candidates.

*M. hominis* CDS		Closest homolog				
Mnemonic	Predicted function	Accession number or mnemonic	Species	% Similarity	Highest similarity in Hominis group %	Comment
**MHO_0350**	Aminopeptidase C	O69192	*Listeria monocytogenes*	59	no^a^	contains motif COG3579, PepC, aminopeptidase C
**MHO_0930**	Diadenosine 5′5′″-P1,P4-tetraphosphatepyrophosphohydrolase (MutT/nudix family protein)	EDP68279	*Carnobacterium sp.*	66	no	no homolog in mycoplasmas
**MHO_1490**	Conserved hypothetical protein	Q48SX1	*Streptococcus pyogenes*	66	no	homologs in several firmicutes from the microbiota
**MHO_2540**	N-dimethylarginine dimethylaminohydrolase	ZP_03894500	*Eggerthella lenta*	76	no^a^	predicted N-dimethylarginine dimethylaminohydrolase in *M. penetrans*, but very different from others
**MHO_4710**	Virulence-associated protein D	O05728	*Helicobacter pylori*	60	no^a^	contains motif VapD, uncharacterized virulence-associated protein D, no homolog in mycoplasmas
**MHO_0360**	Cytosine-specific DNA-methyltransferase/Type II site-specific deoxyribonuclease	MSC0216	*M. mycoides* subsp. *mycoides* SC	83	47	Highly related orthologs in *Clostridium* (83% similarity)
**MHO_0120**	Type III restriction enzyme	UU476	*U. parvum*	71	57	
**MHO_0130**	Pseudogene of Type III restriction-modification system: methylase (part 2)	UU477	*U. parvum*	57	76	probable phase variation, split in *U. parvum*, inferred by synteny
**MHO_0140**	Pseudogene of Type III restriction-modification system: methylase (part 1)	UU478	*U. parvum*	76	73	probable phase variation, split in *U. parvum*, inferred by synteny
**MHO_2520**	Pseudogene of transposase for insertion sequence element IS1138, (part 1)	UU374	*U. parvum*	95	no	truncated
**MHO_2530**	Pseudogene of transposase for insertion sequence element IS1138, (part 2)	UU372	*U. parvum*	94	66	truncated
**MHO_2560**	Truncated integrase-recombinase protein	UU372	*U. parvum*	96	67	truncated
**MHO_3120**	ATP synthase beta chain	UU054	*U. parvum*	92	86	
**MHO_3130**	ATP synthase alpha chain	UU053	*U. parvum*	86	80	
**MHO_3140**	Conserved hypothetical protein	UU052	*U. parvum*	69	46	3 TMB^b^ predicted
**MHO_3150**	Conserved hypothetical protein	UU051	*U. parvum*	68	57	
**MHO_3160**	Conserved hypothetical protein	UU050	*U. parvum*	82	69	
**MHO_3170**	Conserved hypothetical protein	UU049	*U. parvum*	69	61	
**MHO_3180**	Conserved hypothetical protein	UU048	*U. parvum*	72	51	12 TMB predicted
**MHO_3190**	Conserved hypothetical protein	UU046	*U. parvum*	72	63	1 TMB predicted
**MHO_3200**	Conserved hypothetical protein, predicted lipoprotein	UU045	*U. parvum*	65	54	predicted lipoprotein
**MHO_3220**	Type I restriction enzyme specificity protein	UU096	*U. parvum*	56	46	split in *U. parvum*
**MHO_3230**	Type I restriction enzyme specificity protein	UU099	*U. parvum*	45	43	truncated in *U. parvum*
**MHO_3240**	Pseudogene of Type I restriction enzyme M protein (C-terminal part)	UU098	*U. parvum*	81	77	split in *U. parvum*
**MHO_3250**	Pseudogene of Type I restriction enzyme M protein (N-terminal part)	UU098	*U. parvum*	75	81	split in *U. parvum*
**MHO_5210**	Type I restriction enzyme M protein	UU098	*U. parvum*	74	80	split in *U. parvum*
**MHO_5220**	Type I restriction enzyme specificity protein	UU096	*U. parvum*	62	46	split in *U. parvum*
**MHO_5230**	Type I restriction enzyme specificity protein	UU099	*U. parvum*	89	50	truncated in *U. parvum*
**MHO_5240**	Type I restriction-modification system endonuclease	UU095	*U. parvum*	65	70	inferred by synteny

a Except in the phylogenetically closest related *M. arthritidis*.

b TMB, transmembrane segment.

Most of the 59 CDSs showing BBHs in mollicutes belonging to groups other than the Hominis phylogenetic group also had closely related homologs within the Hominis group and phylogenetic reconstructions did not allow clarification of their origin. Nevertheless, features indicative of HGT were found for six genes or groups of genes. In particular, MHO_0360 encoded a predicted type II cytosine-specific methyltransferase, for which the only homolog in mollicutes was found in *M. mycoides* subsp. *mycoides* SC (Table 2). Closely related homologs were also identified in other bacteria, such as *Clostridium* and *Streptococcus* spp., with more than 80% amino-acid sequence similarity. This suggested that a type II restriction/modification system may have been exchanged between these bacteria.

Five clusters of genes from the *M. hominis* genome were found to have their closest homologs in the urogenital pathogen *U. parvum*, which belongs to the phylogenetically distant Pneumoniae group (Table 2). The first cluster included MHO_0120 to MHO_0140, encoding a predicted type III restriction/modification system (Figure S1). Closely related homologs were found in *U. parvum*, *M. penetrans* and several species of the Hominis group. Phylogenetic reconstruction, inferred from restriction enzyme sequences, associated the *M. hominis* (MHO_0120) and *U. parvum* (UU476) homologs with a bootstrap support value of 100%, suggesting that HGT occurred between the two species. In both species, the modification methylase-encoding gene was split into two by a frameshift that occurred within tracts of repeated nucleotides. In MHO_0130/MHO_0140, a simple deletion of one nucleotide within a tract of 11 guanosines restored the complete ORF, suggesting that the expression of the encoded methylase may be switched on and off by a phase variation mechanism, similar to that previously described for another type III restriction/modification system in *M. pulmonis* [18].

The two gene clusters, MHO_5210 to MHO_5240 and MHO_3220 to MHO_3250, encoded predicted type I restriction/modification systems (Figure S2). The first cluster contained full-length *hsdR*, *hsdS* (2 genes) and *hsdM* genes and encoded the components of the system. In contrast, the second cluster only contained the two *hsdS* genes and an *hsdM* gene that was split into two by a frameshift mutation. Nucleotide sequences of the genes within the two clusters were nearly identical, indicative of a probable duplication event. Phylogenetic trees inferred from the protein sequences of the *M. hominis* R and M subunits showed a close relationship with their homologs in *U. parvum* (UU095 to UU100).

A fourth cluster included CDSs MHO_2520 and MHO_2530, two fragments of a transposase-encoding gene from an IS1138 element (Figure S3). Phylogenetic analysis demonstrated a stronger relationship between this gene and its fragmented homolog in *U. parvum* (UU374/UU373/UU372) than with any other transposase gene. Moreover, sequence alignments revealed more than 90% similarity for the predicted proteins and extensive regions in which the nucleotide sequences were strictly identical. These data strongly suggest recent exchange of this mobile element between the two species.

The fifth group of genes that may have been exchanged with *U. parvum* encoded seven conserved hypothetical proteins and two additional ATP synthase subunits (MHO_3120 to MHO_3200) (Figure S4). As described for the other clusters, this potential gene exchange was deduced from protein-inferred phylogenetic trees and conservation of gene order. The seven CHPs were predicted to include one lipoprotein and three proteins with transmembrane segments, suggesting a location at the cell surface.

It should be noted that, whereas five clusters of genes may have been exchanged with the *U. parvum*, no HGT was predicted with the urogenital pathogen *M. genitalium*, or with *M. penetrans*, another human mollicute reported in the urogenital tract [1].

### Comparative analysis of the *M. hominis*, *M. genitalium*, and *U. parvum* genomes

**Figure 1:**
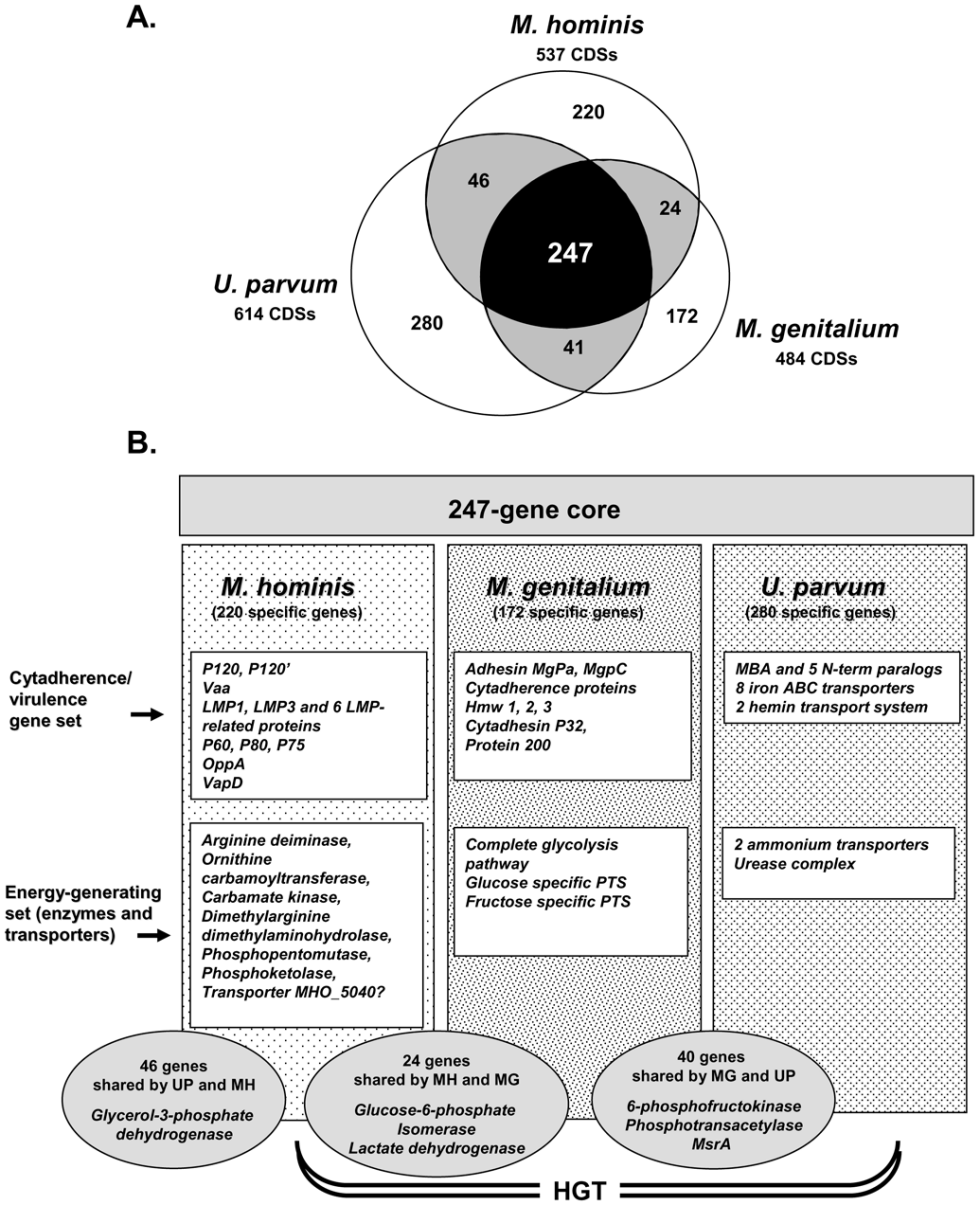
Comparative analysis of the genomes of three minimal mollicutes affecting humans. (A) Number of specific and shared CDSs for the genomes of *M. hominis* PG21, *M. genitalium* G37, and *U. parvum* serovar 3. (B) Focus on known species-specific proteins involved in cytadherence/virulence and energy-generating pathways. LMP, large membrane protein; PTS, phosphoenolpyruvate phosphotransferase system; HGT, horizontal gene transfer; UP, *U. parvum*; MH, *M. hominis*; MG, *M. genitalium*.

The *M. hominis* genome was compared to the genomes of *M. genitalium* and *U. parvum*, the two other urogenital mollicute species that infect humans. Orthology relationships between the three genomes were computed using a bidirectional best hit method. Results are shown in Figure 1A, with the complete analysis provided in Table S1. We found 247 CDSs common to all three genomes, forming the core genome, and identified 220 CDSs specific to *M. hominis*, 172 CDSs specific to *M. genitalium* and 280 CDSs specific to *U. parvum*. The *M. hominis* genome shared 24 CDSs only with *M. genitalium* and 46 CDSs only with *U. parvum*, whereas 41 CDSs found in both *M. genitalium* and *U. parvum* were not found in the *M. hominis* genome.

### The *M. hominis*, *M. genitalium*, and *U. parvum* genomes share a core set of 247 genes, many of which are essential

The 247 orthologous genes shared by the three mollicutes encoded proteins involved in major cellular functions, including DNA metabolism, protein synthesis, nucleotide synthesis, transport and binding of substrates, and fatty acid and phospholipid metabolism (Table S1). Interestingly, these core genes were found to encode only a few cell envelope proteins: the putative membrane conserved hypothetical proteins MHO_3900, MHO_4290 and MHO_4370. Most of these 247 shared genes are likely to be essential, consistent with findings from an extensive gene inactivation study in *M. genitalium* [6]. Indeed, 213 of the 247 genes (86.2%) could not be inactivated by transposon mutagenesis. Moreover, only 35 of these 213 genes could be inactivated in the two phylogenetically-related species *M. arthritidis* [13] or *M. pulmonis* [19] (Table S2). Among the 247 shared genes, 217 were also predicted in the genomes of other human and animal mycoplasmas (Table S2). Most of the 30 remaining genes were only missing in one or two species, suggesting a recent loss. Finally, a total of 160 genes that could not be inactivated were shared by all available mycoplasma genomes.

### Genes related to cytadherence and virulence are mainly species-specific

Many of the species-specific genes were associated with cytadherence and virulence (Figure 1B). Genes found specifically in *M. hominis* coded for: (i) the lipoprotein P120 (MHO_3660) and the protein P120' (MHO_3800), which are *M. hominis*-specific surface-exposed proteins displaying antigenic variation [20],[21]; (ii) the Vaa surface lipoprotein adhesin (MHO_3470), which is an abundant surface antigen displaying high-frequency phase and size variation and which is involved in adhesion to host cells [21],[22]; (iii) the P60 and P80 proteins (MHO_3490, MHO_3500), which form a membrane complex at the surface and are encoded by an operon [23]; (iv) the P75 lipoprotein (MHO_3720), which is also present at the surface of *M. hominis* [24], and the P75-related lipoprotein (MHO_3100), which shows 37.9% similarity with the P75 protein. CDSs specific to *M. hominis* also included OppA (MHO_1510), an oligopeptide permease substrate-binding protein. OppA is a multifunctional lipoprotein involved in cytadherence, but is probably also the main ecto-ATPase at the surface of the *M. hominis* cell [25],[26]. A recent study showed that it induced ATP release from cells, resulting in apoptosis, thus suggesting its role as a *M. hominis* virulence factor [27]. Lmp1 (MHO_0530) and Lmp3 (MHO_1640), two related surface membrane proteins [28], and six additional Lmp-related proteins (MHO_0540, MHO_3070, MHO_3110, MHO_3730, MHO_4280 and MHO_4920), were also found in this group of *M. hominis*-specific genes. The eight Lmp proteins represent a family of proteins characterized by a predicted N-terminal transmembrane helix and several repeat sequences. In particular, using the MEME method, we identified a 57 amino-acid motif, [QK][QK]L[DKQ][DN]LI[DK]S[NQE][DE][AG][KQ][DKN][VI]D[KT][SQ]K[EA][TN][QDN][IS][LF][NQ]N[TN]N[LVI][DT][AG][SKN][SD][LT][IT][KD][DQ]I[EKV][SN][KA][TI][KN][TE]I[EK][DK]A[IT][QEK][SD]L[TQ]K[KL]I[ND], repeated between one and 15 times, in all proteins except MHO_0530 and MHO_4280.

Similarly, genes specific to *M. genitalium* and *U. parvum* genomes included several CDSs associated with cytadherence and virulence (Table S1, Figure 1B). The 172 *M. genitalium* specific CDSs included genes encoding the adhesin MgPa (MG191), the MgpC protein (MG 192), the adhesin P32 (MG318), the protein P200 (MG386) and the cytadherence accessory proteins Hmw1, Hmw2 and Hmw3 (MG218, MG312, MG317). Of the 280 CDSs specific to *U. parvum*, genes associated with cytadherence and virulence included those encoding the MBA protein (UU375) — a major antigen recognized during infection of humans — and the five MBA N-terminal paralogs (UU172, UU189, UU483, UU487, UU526). Genes encoding eight iron transporters (UU022, UU023, UU025, UU357, UU358, UU400, UU515, UU516), nine putative ABC substrate-binding protein-iron (UU024, UU027, UU028, UU069, UU071, UU359, UU360, UU401, UU517) and two hemin transporters (UU070, UU399) were also found in the *U. parvum* genome, but not in *M. hominis* or *M. genitalium*. It should be noted that the virulence-related gene *msrA*, encoding the peptide methionine sulfoxide reductase, was found in both *M. genitalium* (MG408) and *U. parvum* (UU289), but not in *M. hominis*. In *M. genitalium*, inactivation of the *msrA* gene reduces adherence and abolishes virulence [29].

### *M. hominis*, *M. genitalium*, and *U. parvum* display marked species differences in their reduced energetic metabolism

Several species-specific genes were involved in the major energy-generating metabolic pathways. In *M. genitalium*, which is a glycolytic species, several genes encoding components of the carbohydrate, pyruvate and glycerol metabolic pathways were found among its 172 specific CDSs, including carbohydrate transporter genes (Table S1). Indeed, the 1-phosphofructokinase-encoding gene *fru*K (MG063), *fruA* (MG062) and *pstG* (MG069) genes of the fructose and glucose phosphoenolpyruvate-dependent sugar phosphotransferase transport system (PTS) were found specifically in *M. genitalium*. Moreover, the four genes encoding the pyruvate dehydrogenase complex, *pdhA* (MG274), *pdhB* (MG273), *pdhC* (MG272) and *pdhD* (MG271), the glycerol kinase gene *gpl*K (MG038) and the glycerol uptake facilitator gene *glpF* (MG033) were predicted in the *M. genitalium* genome only, and not in the *M. hominis* or *U. parvum* genomes. In *U. parvum*, which generates ATP through urea hydrolysis, the seven components of the urease complex (UU428 to UU434), the ammonium transporters Amt-1 and Amt-2 (UU218, UU219) and the atypical delta subunits of the FoF1-ATPase (UU133, UU134) probably involved in this unique energy production pathway [11], were found among the 280 CDSs that were absent from *M. hominis* and *M. genitalium* (Table S1).

**Figure 2:**
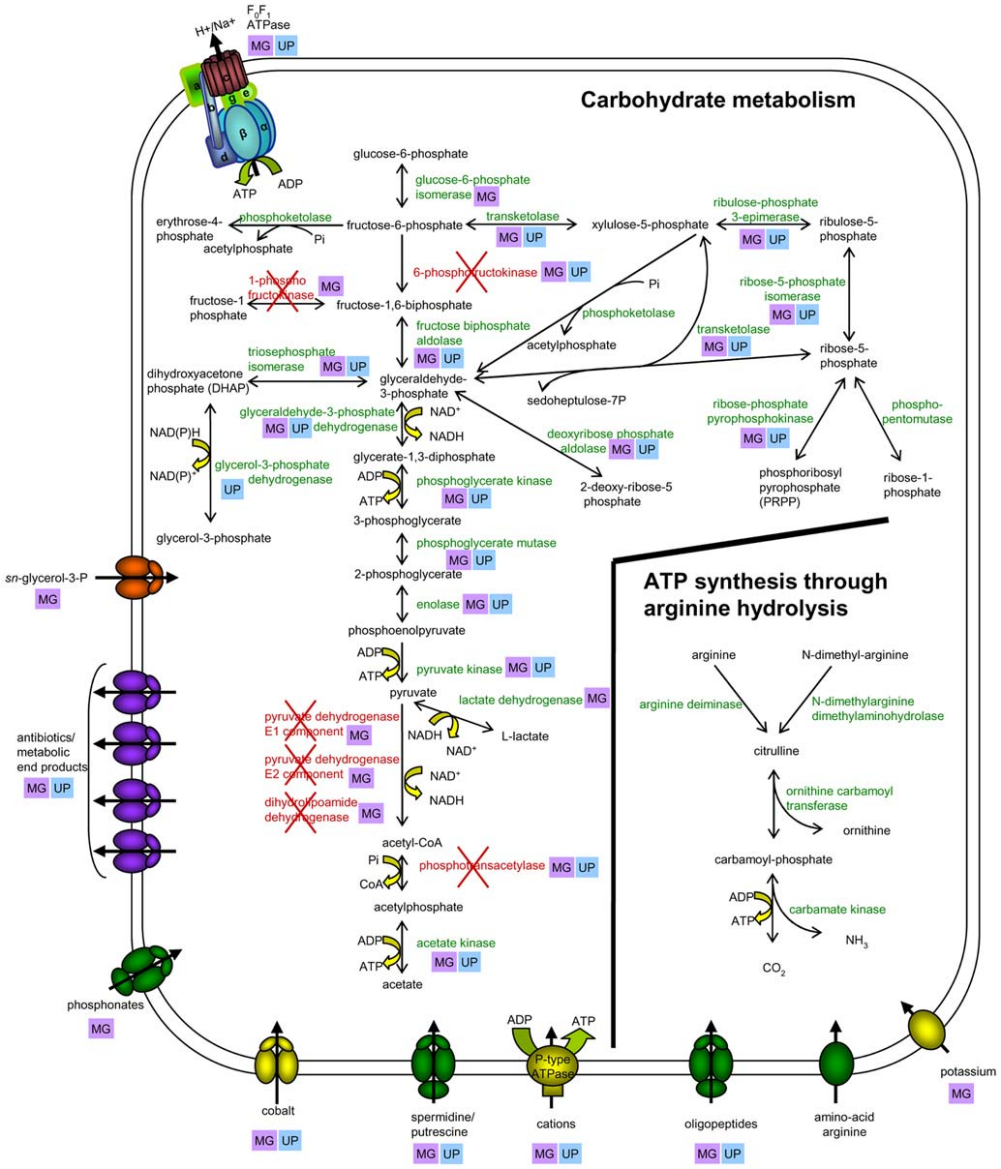
Comparison of carbohydrate- and arginine-metabolism pathways and transporters encoded by *M. hominis* PG21, *M. genitalium* G37, and *U. parvum* serovar 3. Metabolic products are shown in black. Putative proteins present in *M. hominis* are shown in green; proteins absent from *M. hominis* are shown in red. “MG” in purple boxes indicates that the gene encoding the corresponding protein is present in the *M. genitalium* G37 genome. “UP” in blue boxes means that the gene encoding the corresponding protein is present in the *U. parvum* genome. Transporters are colored according to their substrates: yellow, cations; green, anions and amino-acids; orange, carbohydrates; purple, multidrug and metabolic end product efflux. Arrows indicate the direction of substrate transport.

In *M. hominis*, a nonglycolytic species, all the genes coding for all enzymes of the Embden-Meyerhoff-Parnas (EMP) pathway were present except 6-phosphofructokinase (Figure 2). The nonglycolytic *U. parvum*, however, had the 6-phosphofructokinase gene, but did not have the gene encoding glucose-6-phosphate isomerase, which catalyzes the step leading to fructose 6-phosphate production [11] (Figure 2). The set of genes coding for enzymes in the pentose phosphate pathway was incomplete in *M. hominis*, with the absence of genes encoding glucose-6-phosphate dehydrogenase and 6-phosphogluconate dehydrogenase. These genes were also absent from *M. genitalium* and *U. parvum*. The genes coding for other enzymes involved in carbohydrate metabolism were present in *M. hominis* and *U. parvum*, with the exception of 1-phosphofructokinase, which is involved in fructose metabolism, and the pyruvate dehydrogenase complex, as mentioned above [11],[30]. Thus, a complete set of genes encoding components of the EMP pathway was present in *M. genitalium* [3],[30], which is able to generate ATP through glucose hydrolysis, whereas the *M. hominis* and *U. parvum* genomes each lacked a single gene encoding a component of the EMP pathway. It should also be noted that *M. hominis*, like *U. parvum* [11], does not seem to possess a complete phophoenolpyruvate phosphotransferase system (PTS), as only a putative energy-coupling protein HPr (MHO_0590) and the component B of the enzyme II complex (MHO_1070) were predicted. In contrast, the glycolytic *M. genitalium* possessed two complete glucose and fructose PTS. Finally, the two genes encoding enzymes of the acetate kinase–phosphotransacetylase pathway, which is generally fully reversible (acetate+ATP+CoA↔acetyl-CoA+ADP+P_i_) in bacteria, were present in *M. genitalium* and *U. parvum*; but the phosphotransacetylase gene was absent from the *M. hominis* genome (Figure 2, Table S1). Interestingly, glycerol 3-phosphate dehydrogenase (*gpsA*, MHO_1690) was present in both *M. hominis* and *U. parvum*. This enzyme could lead to glyceraldehyde 3-phosphate production via the second part of the EMP pathway involving triosephosphate isomerase (Figure 2).

In *M. hominis* PG21, the three proteins constituting the arginine dihydrolase pathway (Figure 2) — arginine deiminase (ADI) (*arcA*, MHO_0690), ornithine carbamoyltransferase (*arcB*, MHO_0640) and carbamate kinase (*arcC*, MHO_0630) — were found to be specific among the three species discussed here. Moreover, a putative dimethylarginine dimethylaminohydrolase (DDAH, MHO_2540) was predicted in *M. hominis* but not in *U. parvum* and *M. genitalium*. In *P. aeruginosa*, DDAH (EC 3.5.3.18) catalyzes the conversion of the N^ω^-alkylated arginine residues to citrulline. A more detailed overview of the *M. hominis* energy-generating pathway was obtained by searching for specific transporter genes in the genome. The results are graphically displayed in Figure 2. A putative arginine transporter, MHO_5040, containing 12 transmembrane domains was identified as a homolog of the amino-acid permease MYPE6110, a protein which is encoded immediately downstream from the arginine dihydrolase pathway cluster, *arc*ABC, in the *M. penetrans* genome [31], another arginine-utilizing mycoplasma. However, in *M. hominis*, MHO_5040 was not located in close proximity to the *arcABC* cluster. In addition, it should be noted that no ammonia/ammonium transporters was identified in the *M. hominis* genome.

To further determine the major energy-generating pathway in *M. hominis*, we studied the growth of *M. hominis* PG21 in the presence of arginine and arginine analogs (Table 3). In Hayflick modified medium containing 2.5% horse serum, the *M. hominis* culture reached 10^7^ color changing units (CCU)/ml and the pH increased from 7.0 to 7.2 after 48 hours. In medium supplemented with 10 mM arginine, the titer after 48 hour growth was unchanged, but the pH was higher than that observed without addition of arginine, increasing from 7.0 to over 8.1. A total loss of viability was observed after 120 hours under these conditions. The effect on growth of adding horse serum was checked by inoculating media with or without 2.5% horse serum. Growth was independent of serum content in medium supplemented with 10 mM arginine. However, a non-supplemented medium lacking horse serum was growth-limiting, with a 10-fold lower titer of organisms after 48 hours (data not shown). Thus, the serum-containing Hayflick modified medium supplied the cells with nutrients that were necessary to reach a high cell titer after 48 hours. Furthermore, in the absence of horse serum, the exogenously added arginine provided energy for cell growth. The effect on growth of canavanine, an arginine analog that interferes with the arginine uptake and metabolism was evaluated. Canavanine is an ADI-specific competitive inhibitor [32] and its incorporation into proteins in bacterial species leads to cell death [33]. In the presence of 10 mM canavanine, the cell concentration remained constant at 10^5^ CCU/ml over 120 hours. Addition of 23 mM arginine at 48 hours was sufficient to displace the competitive inhibitor and restore growth (Table 3). Canavanine suppressed the cell growth but did not trigger the *M. hominis* death within 120 hours. Thus, while a possible incorporation of canavanine into proteins cannot be totally ruled out, the bacteriostatic effect of canavanine was most probably due to its inhibitory effect on ADI. These data suggest that the ADI pathway was required for initiation of growth in *M. hominis*. Finally, to test whether asymmetric N^ω^,N^ω^-dimethyl-L-arginine (ADMA) could be used as a substrate by DDAH and could serve as an energy source for protein synthesis in *M. hominis*, growth was evaluated in the presence of ADMA with ADI activity suppressed by canavanine. The maximum concentration of ADMA that could be assessed was 0.5 mM, because of the poor solubility and dissolution rate of the arginine analog in aqueous solutions. Under these conditions, in presence of 2.5% horse serum in the medium (Table 3), as well in medium lacking horse serum, no growth was detected after 48 hours.

**Table 3:** Growth of *M. hominis* PG21 in the presence of arginine and arginine analogs.

*M. hominis* in basal medium^a^ supplemented with:	Growth in CCU/ml^b^ (pH)		
	t = 0	t = 48 h	t = 120 h
No supplement	10^5^ (7.0)	10^7^ (7.2)	10^6^ (7.4)
Arginine (10 mM)	10^5^ (7.0)	10^7^ (8.1)	0 (8.2)
No supplement + Arginine (23 mM) at t = 48 h	10^5^ (7.0)	10^7^ (7.2)	10^5^ (7.9)
Canavanine (10 mM)	10^5^ (7.0)	10^5^ (6.7)	10^5^ (6.8)
Canavanine (10 mM) at t = 0 + arginine (23 mM) at t = 48 h	10^5^ (7.0)	10^5^ (6.7)	10^7^ (7.2)
Canavanine (10 mM) +ADMA^c^ (0.5 mM)	10^5^ (7.0)	10^5^ (6.9)	ND^d^

a containing heart infusion broth, yeast extract and 2.5% horse serum.

b CCU/ml, color changing unit/ml.

c ADMA, *N^ω^*,*N^ω^*-dimethyl-L-arginine.

d ND: not determined.

## Discussion

In this study, we first sequenced, annotated and analyzed the genome of *M. hominis* PG21. Our analyses suggested the occurrence of HGT between *M. hominis* and *U parvum*. Indeed, five clusters of genes from the *M. hominis* genome were found to have their closest homolog in *U. parvum*, which belongs to the phylogenetically distant Pneumoniae group. These mollicutes share the same urogenital niche, providing a potential explanation for the predicted HGT. Phylogenomic studies have recently reported the occurrence of HGT among animal mycoplasmas sharing the same host. Indeed, 18% of the *M. agalactiae* genome has undergone HGT with other pathogenic, ruminant mycoplasmas from the phylogenetically distant Mycoides cluster [34]. In *M. hominis*, the potential advantages of these events remain unclear. However, the largest gene cluster predicted to have undergone HGT (MHO_3120 to MHO_3200) contains four putative cell-surface proteins that may be involved in the interaction between the mycoplasma and its human host. This is the first report of HGT among human mollicutes. We did not find any evidence of HGT between *M. hominis* and *M. genitalium*, or *M. penetrans*. *M. hominis* and *U. parvum* are both frequent commensals of the human urogenital tract and they are commonly detected together in healthy individuals. In contrast, *M. penetrans* is very rarely detected in the urogenital tract [1], and the commensal status of *M. genitalium*, which has a low prevalence in the general population [35], remains unclear. Overall, these data suggest that genome reduction was not the only factor affecting the evolution of these minimal organisms.

We also compared the whole genomes of the three human pathogenic mycoplasmas. Two major sets of genes could be identified among species-specific genes. The first group contained genes involved in energy-generating mechanisms, with the second group containing genes involved in cytadherence and virulence. Thus, these three mycoplasma species have their own specific genetic equipment underlying their pathogenic roles. We found several CDSs specific to *M. hominis*. These included the genes encoding P120, P120', Vaa, LMP1 and LMP3, which have been previously identified as surface proteins displaying size, sequence, and antigenic variation [20]–[22], [24]–[26],[28], and six genes annotated as LMP-related proteins. The large number of members of the LMP-related protein group and the repetitions present in their DNA sequences is likely to provide the basis for genetic variability, potentially playing a significant role in determining the response to host defense mechanisms. Parallels can be drawn with the multiple complete or partial copies of the *M. pneumoniae* P1 adhesin gene [36] or the *M. genitalium mgpB* adhesin gene [3] dispersed on the chromosome, which serve as reservoirs to generate antigenic variation and participate in host defense evasion [37],[38]. Proteomic studies could be used to identify the LMP-related proteins expressed in *M. hominis* to further elucidate the role of LMPs. Size variation among these cell envelope proteins could also explain the heterogeneity in genome size within the *M. hominis* species. Indeed, genome size was previously estimated for 15 strains using pulsed-field gel electrophoresis, and was found to vary by more than 15%, ranging from 696 to 825 Kbp [10]. It is likely that there are a number of genes involved in *M. hominis* pathogenic mechanisms that are yet to be described, so the characterization of genes specific to *M. hominis* could help to identify unknown adhesion or virulence factors. Indeed, such factors could be searched for among the 220 *M. hominis*-specific CDSs, particularly among the hypothetical proteins, although virulence factors may also be shared with other mycoplasma species.

*M. hominis* has been described as a nonglycolytic species that generates ATP through arginine hydrolysis [12],[39], as previously described for *M. arthritidis*, the mycoplasma most closely related to it for which the genome sequence is also available [12]. The question of whether arginine degradation was the sole or the major energy-generating mechanism has been asked for more than 30 years. We thus analyzed the genomes of the three mycoplasmas for their genes encoding enzymes involved in the energy generation from carbohydrate metabolism. Analysis of the *M. hominis* genome revealed that the gene sets of the pentose phosphate pathway and of the glucose and fructose transport PTS were incomplete, as has been observed for other sequenced glycolytic and nonglycolytic mollicutes [30],[39],[40]. Furthermore, previous enzymatic activity studies, reporting the absence of 6-phosphofructokinase activity, from the EMP pathway, in seven different strains of *M. hominis* were confirmed *in silico* by the lack of the corresponding gene [12]. Thus, as previously proposed for *U. parvum* [11],[30], it is unlikely that *M. hominis* metabolizes hexoses such as glucose or fructose. The presence of a gene encoding glycerol 3-phosphate dehydrogenase in *M. hominis* suggests that glycerol 3-phosphate may be oxidized into dihydroxyacetone phosphate, which could then enter glycolysis (Figure 2). This would require glycerol or glycerol 3-phosphate to be efficiently imported into the cell. This transport could be mediated by a specific ABC transporter for *sn*-glycerol phosphate, putatively encoded by MHO_0740–0760. Indeed, MHO_0740–0760 shows a BBH and conserved synteny with genes encoding putative glycerol transporters in other mollicute species, such as *M. synoviae* and *M. pneumoniae* [36],[41]. Further downstream in this pathway, energy is not generated through oxidation of pyruvate to acetyl-CoA, since the genes coding for the pyruvate dehydrogenase complex are absent from the *M. hominis* genome (Figure 2). The reversible acetate kinase-phosphotransacetylase pathway was predicted to be incomplete in *M. hominis*, suggesting that the putative conversion of acetate into acetylphosphate does not lead to generation of acetylCoA. Nevertheless, the presence of the gene encoding acetate kinase (*ack*A, MHO_3840) is particularly notable, given the possible role of this enzyme in ATP generation in *M. hominis* [42]. Acetylphosphate may be produced during the conversion of xylulose 5-phosphate to glyceraldehyde 3-phosphate by phosphoketolase (MHO_3010), the gene for which is in the *M. hominis* genome but is absent from the *U. parvum* and *M. genitalium* genomes. The presence of several gaps in the *M. hominis* glycolysis and pyruvate pathways supports the notion that these energy-generating pathways have a low efficiency in this species. Consistent with this, we observed that the growth of *M. hominis* in Hayflick modified broth supplemented with glucose [43] led to alkalinisation of the culture medium (data not shown). *M. hominis* species-specific genes included a complete set of genes coding for the enzymes of the arginine dihydrolase pathway, which can generate energy in the form of ATP for growth. Considering that alternative energy-yielding pathways have been demonstrated for several arginine-utilizing mollicutes [44],[45], it remains unclear whether the ATP produced by arginine breakdown can satisfy all *M. hominis* growth requirements. Arginine is considered an essential nutrient for initiation of growth of *M. hominis* [46]. However, the lack of correlation between CO_2_ production, cell growth and ADI activity, as well as the delayed induction of ADI relative to the cell cycle, led Fenske and Kenny [47] to suggest that *M. hominis* used the arginine pathway only as an alternative energy source. In this study, we showed arginine to be sufficient for cell growth in a growth-limiting medium. Interestingly, the pH of the medium after 48 hours of growth varied with the initial composition of the medium. Supplementation of the basal medium with arginine resulted in an increase of the pH, probably due to a higher rate of ammonium production via the arginine dihydrolase pathway. This increase in pH could have deleterious effects on cell survival *in vitro*, as suggested by the loss of viability after 120 hours in the presence of arginine. Moreover, canavanine, a specific competitive inhibitor of ADI from *M. arthritidis* [32], suppressed growth of *M. hominis*, suggesting that ADI activity is essential for its growth. For its metabolism, arginine can be transported into *M. hominis* cells in its free-form [46]. In our study, arginine was provided in its free and peptide-bound forms. The free amino-acid may enter the cell via the putative arginine transporter (MHO_5040). Additionally, given that there are genes encoding a putative Opp ABC system (MHO_1510–1550) in the *M. hominis* genome, arginine-containing oligopeptides should be considered as possible alternative source of arginine. Currently, a considerable limitation on *in vivo* metabolic studies is the absence of a defined medium for growing *M. hominis*. An additional *in silico* genome-scale metabolic reconstruction of the metabolic pathways could help to elucidate the true biochemical capacity of *M. hominis*, as recently described for *M. genitalium* [48].

We identified a gene encoding a putative DDAH, an enzyme structurally related to ADI, in the *M. hominis* PG21 genome. This suggests that a secondary citrulline-producing pathway, involving the catalytic conversion of ADMA, may occur in *M. hominis*. However, addition of a maximum soluble concentration of ADMA to a medium supplemented with canavanine was not sufficient to promote detectable growth. The ADMA concentration was probably not high enough in our experiments to allow a significant increase in bacterial cell concentration. Thus, the potential production of additional citrulline through metabolism of ADMA in *M. hominis* remains uncertain but cannot be excluded. ADMA is naturally present in biological fluids and inhibits nitric oxide synthase in humans [49]. As previously reported for *P. aeruginosa* [50], production of DDAH by *M. hominis* could indirectly increase host NO production leading to tissue damage. Consistent with this hypothesis, *M. hominis* can stimulate nitric oxide synthase production by host macrophages [51]. It should also be noted that the DDAH gene found in *M. hominis* and its truncated ortholog found in the closely related *M. arthritidis* may have been acquired by HGT from non-mollicute bacterial species, notably from *A. vaginae* which belongs to the same urogenital niche than *M. hominis* and is also involved in bacterial vaginosis [52].

The arginine utilization pathway may have implications for the relationship between *M. hominis* and its host through ammonia formation. This compound is also generated during the hydrolysis of urea in *U. parvum*. As both species are mainly found in the acidic female genital tract, these pathways could be required to protect them from the deleterious effects of the acidic environment and may also promote their pathogenicity. Moreover, *M. hominis* may be able to form biofilms since it was identified in microbial biofilms involved in intraamniotic infection [53]. The ADI pathway may therefore also play a role in biofilm formation, as previously suggested for *Staphylococcus aureus* [54]. Indeed, in *S. aureus*, the expression levels of the deiminase pathway were higher in biofilm cells than in planktonic cells.

To identify a minimal gene set essential for cell viability, Glass *et al.* used a global transposon mutagenesis strategy in *M. genitalium*. They found 382 of the 482 protein-coding genes to be essential under laboratory conditions [6]. However, when the 537 *M. hominis* protein-coding genes were compared with this set, 147 (38%) were absent from the *M. hominis* genome (data not shown). These 147 genes absent from *M. hominis* mainly encoded proteins involved in carbohydrate transport and metabolism (PtsH, PtsG, PtsI, 6-phosphofructokinase, glycerol kinase, pyruvate dehydrogenase complex) or in cytadherence and virulence (MgPa, MgpC, P32, P200, cytadherence accessory proteins Hmw1, 2 and 3). It is therefore possible that at least two whole gene sets involved in cytadherence/virulence and in energy-generating carbohydrate metabolism may be replaced by two other whole gene sets facilitating similar functions in another minimal species (Figure 1B). Thus, it is possible that a complete set of genes constituting an effective energetic pathway, rather than an isolated gene, should be considered essential. This notion can be extended to the adhesion/virulence gene set, as most of the *M. genitalium* adhesion/virulence genes have been demonstrated to be essential [6]. Consequently, in *M. hominis*, in addition to the 247 core CDSs shared by the three minimal species infecting humans, the energy-generating gene set would mainly consist of the three genes of the arginine deiminase pathway and the DDAH-encoding gene. However, the phosphopentomutase- (MHO_4170) and the phosphoketolase- (MHO_3010) genes, which are specific to *M. hominis*, and three genes absent from the core genome but present in *M. hominis* and in one other species (encoding glucose 6-phosphate isomerase, lactate dehydrogenase and glycerol 3-phosphate dehydrogenase) (Figure 1B) should be added to this set of energy-providing genes, as they may be required for optimal energy metabolism in *M. hominis*. Thus, a minimal mycoplasma cell, not including cytadherence and virulence-related factors, could be envisaged to have the 247 gene core genome plus a set of genes involved in energy production but not already in the core. This set would include at least nine genes in *M. hominis*, given that the genes encoding putative transporters of arginine or sn-glycerol 3-phosphate have not been clearly identified. Thus, in *M. hominis*, this would result in a theoretical minimal genome of 256 (247+9) genes. This gene list could be useful for the synthesis of artificial genomes using the recently reported complete chemical synthesis procedure [7],[8].

In conclusion, *M. hominis* PG21 has the second smallest genome among self-replicating free-living organisms. This species hydrolyses arginine as its major energy-source and possesses its own set of cell-surface proteins as the basis of its urogenital pathogenicity. We have described a 247 gene core genome based on whole genome comparisons between *M. hominis* and the two other human urogenital pathogens with minimal genomes, *M. genitalium* and *U. parvum*. An additional set of nine genes may be required for the energy generation in *M. hominis*. Moreover, a set of 220 *M. hominis*-specific genes were identified, which may harbor unknown virulence genes.

## Materials and Methods

### Mycoplasma strain

The *M. hominis* type strain PG21 (ATCC 23114) was chosen and grown in Hayflick modified medium supplemented with arginine [43]. Genomic DNA was isolated as previously described [55].

### DNA sequencing, sequence assembly, and gap closure

We constructed three libraries to determine the complete sequence of the *M. hominis* PG21 genome. For plasmid libraries, genomic DNA was mechanically sheared and 3 kb fragments were cloned into pNAV(A) and pCNS vectors (B) (derived from pcDNA2.1 and pSU18, respectively). Large inserts (25 kb), generated by *Hin*dIII partial digestion, were introduced into pBeloBac11 (New England Biolabs, Ipswich, USA) (C). Vector DNA was purified and end-sequenced using dye-terminator chemistry on ABI3730 sequencers (13440, 8109 and 2304 reads for library A, B and C, respectively). The Phred/Phrap/Consed software package (www.phrap.com) was used for the sequence assembly. A complement of 91 sequences was needed for gap closure and quality assessment.

### Identification of genetic elements and annotation

The genome annotation was performed using the CAAT-Box platform [56], extended with different tools to facilitate the annotation process, as previously described [34]. Briefly, CDSs were detected with the trained GeneMark software [57], implemented in CAAT-Box, which also integrated results of BLAST searches [58] in three databases — SwissProt (http://www.ebi.ac.uk/swissprot/index.html), TrEMBL (http://www.ebi.ac.uk/embl/index.html), and MolliGen (http://cbi.labri.fr/outils/molligen/). Sequence similarity and start codons were determined as previously described [34]. Predicted proteins with similarity lower than 40% or with only local similarities with previously characterized proteins were annotated as hypothetical proteins. Small CDSs or gene fragments were systematically searched for in intergenic sequences of more than 80 bp with BLASTX. The annotation of each CDS was manually verified by at least two annotators. The tRNA genes were identified as previously described [34] and the rRNA genes were mapped onto the chromosome using previously published sequences [10],[55].

Lipoproteins were predicted using the PROSITE prokaryotic membrane lipoprotein lipid attachment site motif (PROKAR_LIPOPROTEIN).

### Detection of Horizontal Gene Transfers in the *M. hominis* genome

Putative HGTs were identified as previously described [34]. Briefly, BBHs were identified for every predicted protein using a BLASTP threshold E-value of 10^−8^. CDSs displaying a BBH from a species not belonging to the Hominis phylogenetic group were further investigated by phylogenetic analyses. Protein phylogenies were determined using the MEGA4 package [59]. Trees were constructed using the distance/neighbor-joining method and the gap complete deletion option; bootstrap statistical analyses were performed with 500 replicates. Bootstrap values lower than 90% were not considered significant. Incongruence between protein and species phylogenies, supported by significant bootstrap values, was considered to be a potential HGT. Genes for which only very few homologs were identified or with branches only supported by low bootstrap values, were not considered to have undergone HGT unless other independent results suggested otherwise. Independent results indicative of the occurrence of HGT were a particularly high similarity value (>80%) and/or conservation of gene synteny.

### Genome analysis and comparisons

Genome analysis and comparisons were conducted using MolliGen, a database dedicated to the mollicute genomes [60]. Orthologous genes in *M. hominis* PG21, *M. genitalium* G37 and *U. parvum* serovar 3 (ATCC 700970) were determined using the bi-directional best hit method followed by manual curation.

### Growth of *M. hominis* PG21 in the presence of arginine and arginine analogs

*M. hominis* PG21 was grown in a basal medium containing heart infusion broth (Difco), yeast extract (Bio-Rad), and 2.5% horse serum (Difco), with or without 10 mM arginine (Fluka), 10 mM canavanine (Sigma) or 0.5 mM N^ω^,N^ω^-dimethyl-L-arginine (ADMA) (Sigma) in 4% dimethylsufoxide, individually or in combination. Identical experiments were conducted with a basal medium without horse serum to limit the arginine content. The pH of the media was adjusted to 7 before inoculation with *M. hominis*. The initial free arginine concentration of 2.5% serum-containing Hayflick media was measured at 1.4 mM by HPLC (High Performance Liquid Chromatography) after deproteinisation. Incubation time was 48 and 120 hours and growth was measured in CCU per ml [43]. At t = 0, 48 and 120 hours, 100 µl of culture was diluted to generate a tenfold dilution series to 10^−12^ in Hayflick modified broth medium supplemented with 23 mM arginine. Results are the means of at least three independent experiments.

### Database submission and web-accessible database

The genome sequence and related features of the *M. hominis* PG21 strain were submitted to the EMBL/GenBank/DDBJ databases under accession number FP236530. All data are also available from the MolliGen database (http://cbib1.cbib.u-bordeaux2.fr/outils/molligen/home.php).

## Supporting Information

Figure S1 Analysis of the *M.hominis* genomic region MHO_0120-MHO_0140. (A) Schematic of the homologous genomic regions encoding a Type III restriction-modification system in *U. parvum* (UU), *M. hominis* (MHO), and *M. pulmonis* (MYPU). Homologous genes are connected by dashed lines. (B) Partial amino acid and nucleotide multiple alignments corresponding to the C-terminal region of MHO_0140. Repeats that may be involved in phase variation are written in blue; stop codons are in red. (C) Phylogenetic tree inferred from amino acid sequences of the restriction component of the systems. Bootstrap values are indicated on nodes. MAG, *M. agalactiae*; MHP7448, *M. hyopneumoniae* 7448; mhp, *M. hyopneumoniae* 232; MHJ, *M. hyopneumoniae* J; MYPU, *M. pulmonis*; MYPE, *M. penetrans*; MMOB, *M. mobile*. Numbers after the strain name indicate mnemonics.
(0.87 MB TIF)

Figure S2 Analysis of the *M. hominis* genomic regions MHO_3220-MHO_3250 and MHO_5210-MHO_5240 encoding Type I restriction-modification systems. (A) Schematic of the homologous genomic regions in *U. parvum* (UU) and *M. hominis* (MHO). Homologous genes are connected by dashed lines; the locus is recombined in *U. parvum*. Phylogenetic trees inferred from amino acid sequences of the MHO_5230 homologs (S subunit) and MHO_5210 homologs (M subunit) are presented in (B) and (C), respectively. Bootstrap values are indicated on nodes. MPN, *M. pneumoniae*; MAG, *M. agalactiae*; SPICI, *Spiroplasma citri*; MGA, *M. gallisepticum*; MYPU, *M. pulmonis*; MMOB, *M. mobile*. Numbers after the strain name indicate mnemonics.
(0.71 MB TIF)

Figure S3 Analysis of the *M. hominis* genomic region MHO_2520-MHO_2530. (A) Schematic of the homologous genomic regions encoding IS1138 transposase gene in *U. parvum* (UU), *M. hominis* (MHO) and *M. pulmonis* (MYPU). The transposase encoding gene is fragmented in *M. hominis* and *U. parvum*. (B) Partial nucleotide alignment showing the nearly identical sequences of MHO_2530, MHO_2560, and UU372. (C) Phylogenetic tree inferred from amino acid sequences of the IS1138 transposases. Bootstrap values are indicated on nodes. MSC, *M. mycoides* subsp. *mycoides* SC; SPICI, *S. citri*; AYWB, *ca.* Phytoplasma asteris Aster Yellows Witches Broom; PAM, *ca.* Phytoplasma asteris Onion Yellows. Numbers after the strain name indicate mnemonics.
(0.83 MB TIF)

Figure S4 Analysis of the *M. hominis* genomic region MHO_3120-MHO_3200. (A) Schematic of the homologous genomic regions in *U. parvum* (UU) and *M. hominis* (MHO). Homologous genes are connected by dashed lines. Phylogenetic trees inferred from amino acid sequences of the MHO_3120 homologs and MHO_3200 homologs are presented in (B) and (C), respectively. Bootstrap values are indicated on nodes. MSC, *M. mycoides* subsp. *mycoides* SC; MCAP, *M. capricolum* subsp. *capricolum*; MAG, *M. agalactiae*; mhp, *M. hyopneumoniae*; MGA, *M. gallisepticum*; MYPU, *M. pulmonis*; MS53, *M. synoviae*; MMOB, *M. mobile*. Numbers after the strain name indicate mnemonics.
(0.73 MB TIF)

Table S1 Orthology between the genomes of *M. hominis*, *M. genitalium* and *U. parvum*. These data are presented in an excel file to facilitate analysis. Orthology was determined using a bidirectional best hit method, followed by manual curation. The columns are as follows: (A) *M. hominis* gene locus (mnemonic); (B) *M. hominis* gene name; (C) *M. hominis* gene product; (D) *M. genitalium* gene locus (mnemonic); (E) *M. genitalium* gene name; (F) *M. genitalium* gene product; (G) *U. parvum* gene locus (mnemonic); (H) *U. parvum* gene name; (I) *U. parvum* gene product.
(0.21 MB XLS)

Table S2 The 247 genes shared by *M. hominis*, *M. genitalium* and *U. parvum*:transposon insertions in orthologs from *M. pulmonis*, *M. arthritidis*, and *M. genitalium* and orthologs in other human and animal mycoplasma sequenced genomes. The columns are as follows: (A) Gene name, (B) Product, (C) Locus in *M. hominis* PG21 (mnemonic), (D) Locus in *M. genitalium* G37 [3] (mnemonic), (E) Locus in *U. parvum* serovar 3 [11] (mnemonic), (F) Transposon insertion in ortholog from *M. pulmonis* according to [19], (G) Transposon insertion in ortholog from *M. arthritidis* according to [13], (H) Transposon insertion in ortholog from *M. genitalium* according to [6], (I–U) Predicted orthologs in other human and animal mycoplasma sequenced genomes, *M. capricolum* subsp. *capricolum*, *M. mycoides* subsp. *mycoides* SC, *M. gallisepticum*, *M. genitalium*, *M. pneumoniae*, *M. penetrans*, *U. parvum*, *M. agalactiae*, *M. arthritidis*, *M. hyopneumoniae* 232, *M. mobile*, *M. pulmonis*, *M. synoviae*, respectively. (V) Presence in all species.
(0.16 MB XLS)
