# Supplementary figures and images for: Life on Arginine for Mycoplasma hominis: Clues from Its Minimal Genome and Comparison with Other Human Urogenital Mycoplasmas
Source: PLoS Genet. 2009 Oct 9;5(10):e1000677. doi: 10.1371/journal.pgen.1000677 (PMC2751442; doi:10.1371/journal.pgen.1000677)

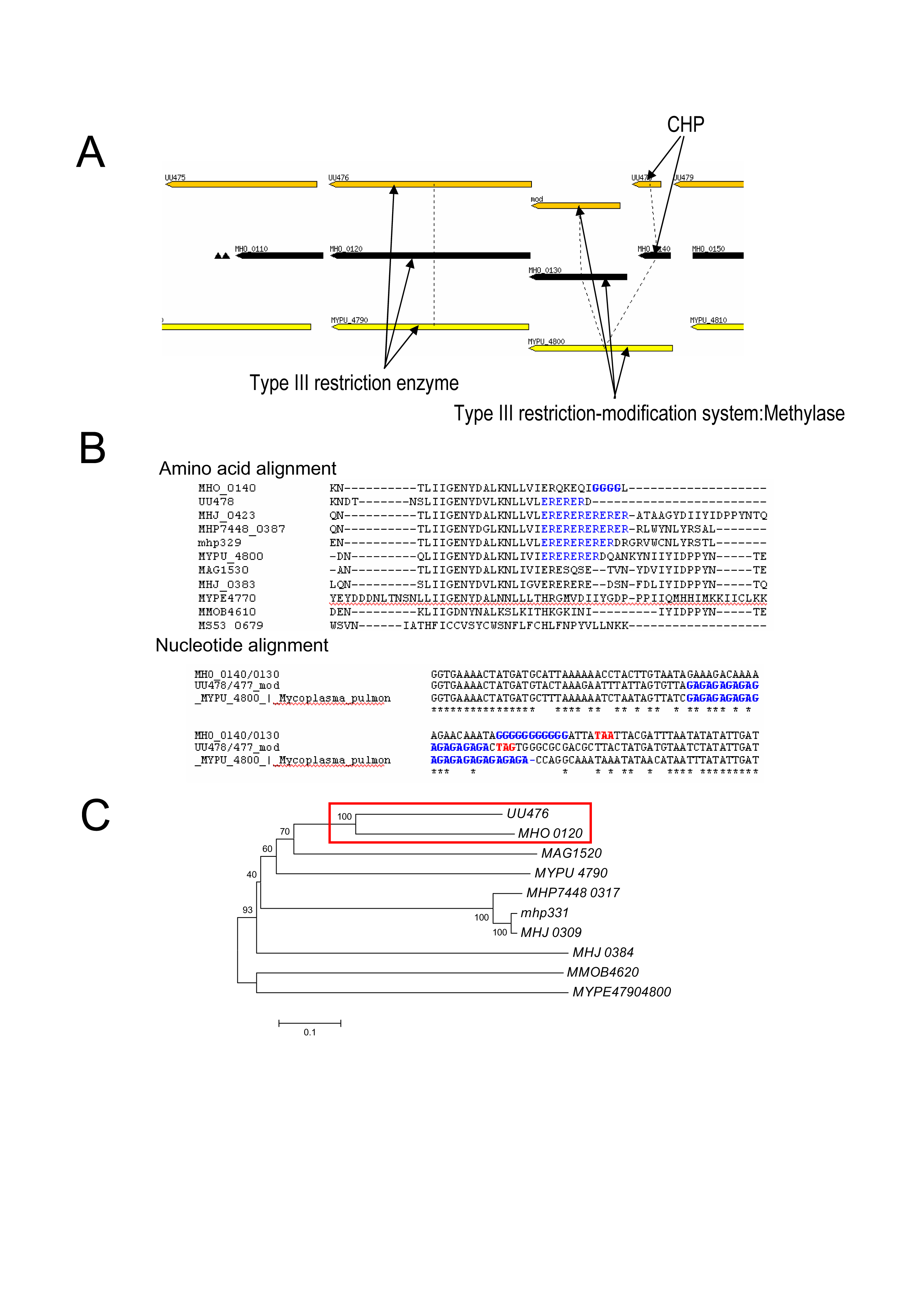

Supplement: Figure S1 — Analysis of the M.hominis genomic region MHO_0120-MHO_0140. (A) Schematic of the homologous genomic regions encoding a Type III restriction-modification system in U. parvum (UU), M. hominis (MHO), and M. pulmonis (MYPU). Homologous genes are connected by dashed lines. (B) Partial amino acid and nucleotide multiple alignments corresponding to the C-terminal region of MHO_0140. Repeats that may be involved in phase variation are written in blue; stop codons are in red. (C) Phylogenetic tree inferred from amino acid sequences of the restriction component of the systems. Bootstrap values are indicated on nodes. MAG, M. agalactiae; MHP7448, M. hyopneumoniae 7448; mhp, M. hyopneumoniae 232; MHJ, M. hyopneumoniae J; MYPU, M. pulmonis; MYPE, M. penetrans; MMOB, M. mobile. Numbers after the strain name indicate mnemonics. (0.87 MB TIF) [file pgen.1000677.s001.tif]

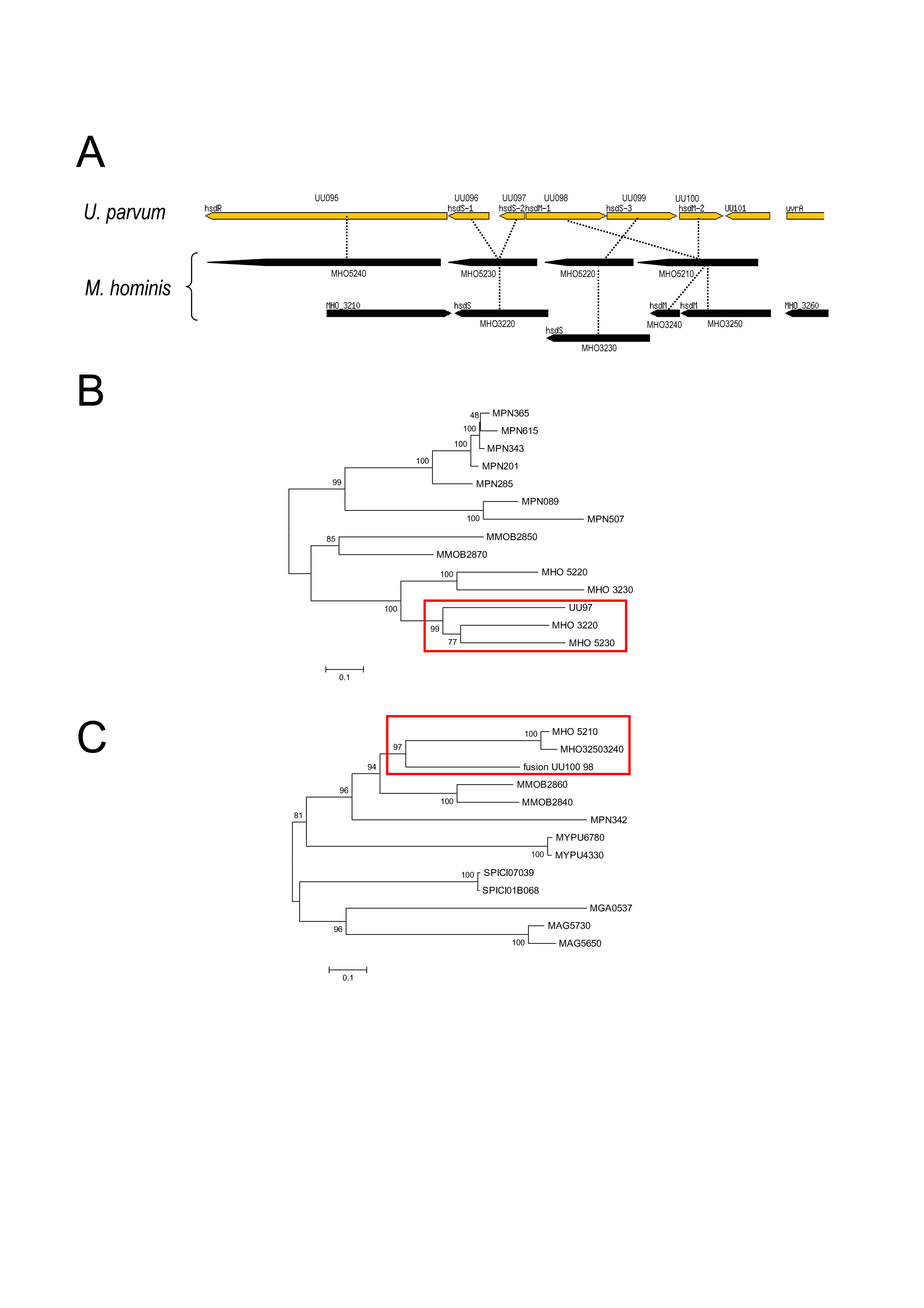

Supplement: Figure S2 — Analysis of the M. hominis genomic regions MHO_3220-MHO_3250 and MHO_5210-MHO_5240 encoding Type I restriction-modification systems. (A) Schematic of the homologous genomic regions in U. parvum (UU) and M. hominis (MHO). Homologous genes are connected by dashed lines; the locus is recombined in U. parvum. Phylogenetic trees inferred from amino acid sequences of the MHO_5230 homologs (S subunit) and MHO_5210 homologs (M subunit) are presented in (B) and (C), respectively. Bootstrap values are indicated on nodes. MPN, M. pneumoniae; MAG, M. agalactiae; SPICI, Spiroplasma citri; MGA, M. gallisepticum; MYPU, M. pulmonis; MMOB, M. mobile. Numbers after the strain name indicate mnemonics. (0.71 MB TIF) [file pgen.1000677.s002.tif]

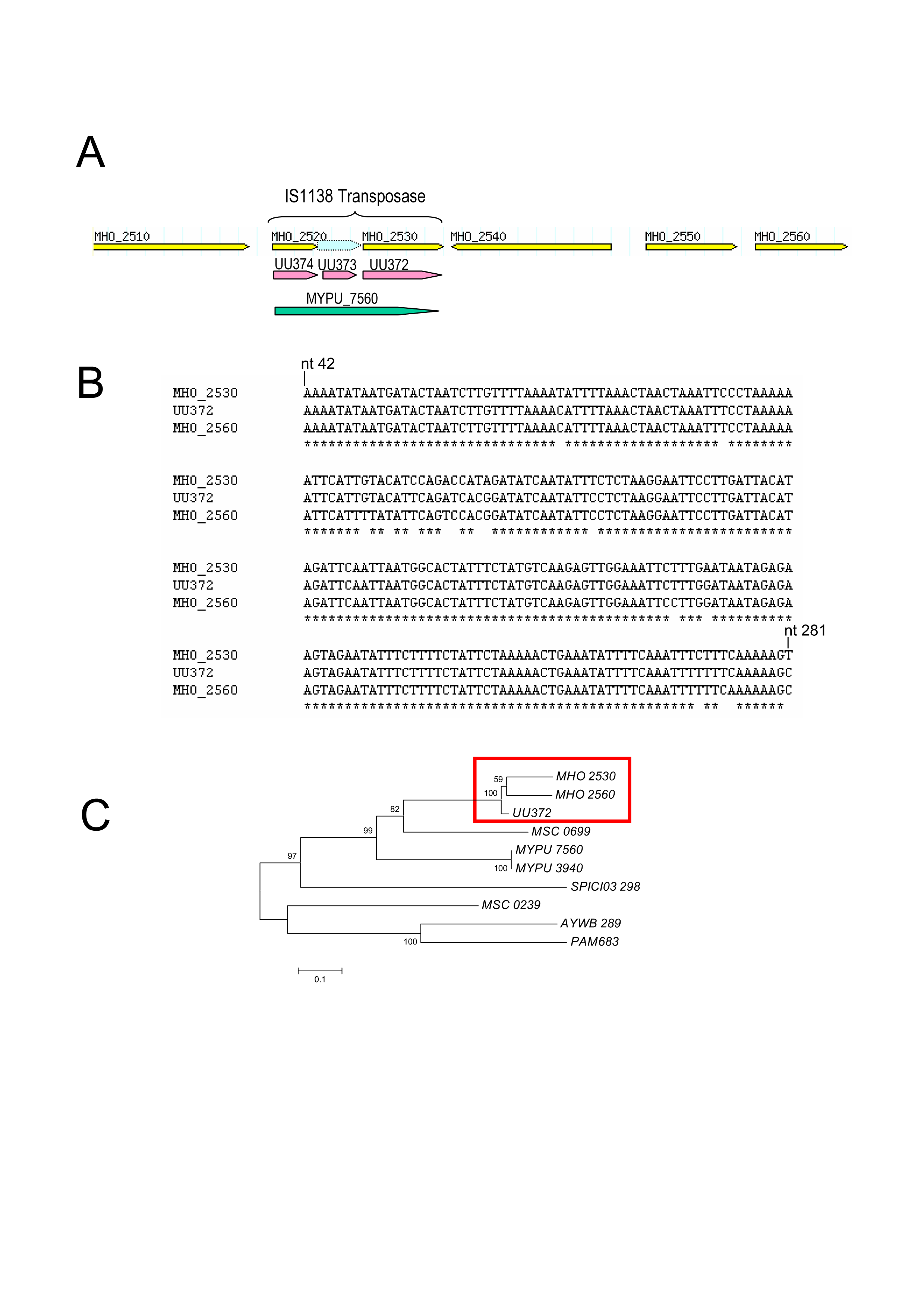

Supplement: Figure S3 — Analysis of the M. hominis genomic region MHO_2520-MHO_2530. (A) Schematic of the homologous genomic regions encoding IS1138 transposase gene in U. parvum (UU), M. hominis (MHO) and M. pulmonis (MYPU). The transposase encoding gene is fragmented in M. hominis and U. parvum. (B) Partial nucleotide alignment showing the nearly identical sequences of MHO_2530, MHO_2560, and UU372. (C) Phylogenetic tree inferred from amino acid sequences of the IS1138 transposases. Bootstrap values are indicated on nodes. MSC, M. mycoides subsp. mycoides SC; SPICI, S. citri; AYWB, ca. Phytoplasma asteris Aster Yellows Witches Broom; PAM, ca. Phytoplasma asteris Onion Yellows. Numbers after the strain name indicate mnemonics. (0.83 MB TIF) [file pgen.1000677.s003.tif]

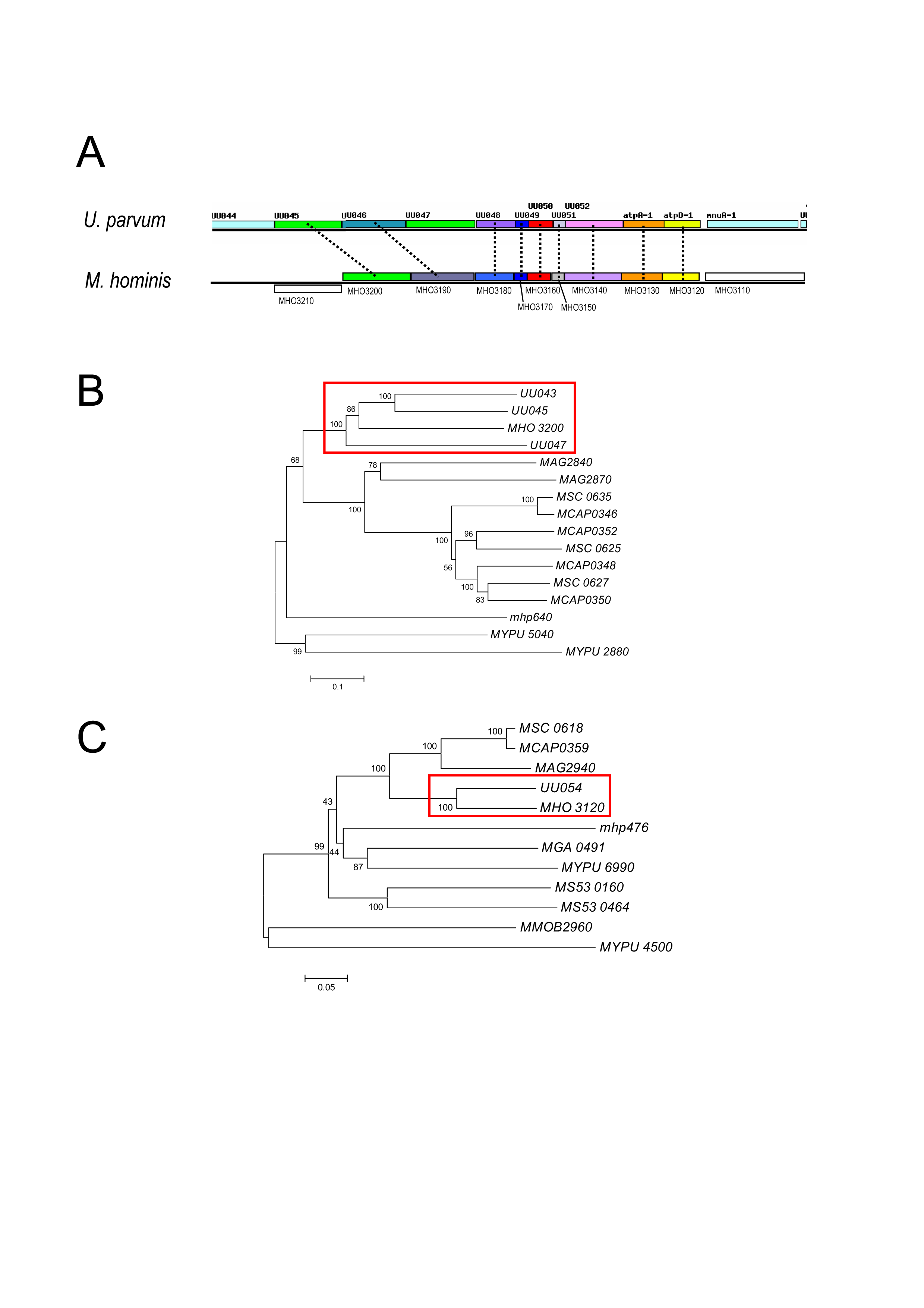

Supplement: Figure S4 — Analysis of the M. hominis genomic region MHO_3120-MHO_3200. (A) Schematic of the homologous genomic regions in U. parvum (UU) and M. hominis (MHO). Homologous genes are connected by dashed lines. Phylogenetic trees inferred from amino acid sequences of the MHO_3120 homologs and MHO_3200 homologs are presented in (B) and (C), respectively. Bootstrap values are indicated on nodes. MSC, M. mycoides subsp. mycoides SC; MCAP, M. capricolum subsp. capricolum; MAG, M. agalactiae; mhp, M. hyopneumoniae; MGA, M. gallisepticum; MYPU, M. pulmonis; MS53, M. synoviae; MMOB, M. mobile. Numbers after the strain name indicate mnemonics. (0.73 MB TIF) [file pgen.1000677.s004.tif]
